# Selection of subjects for clinical trials in Alzheimer’s disease and mild cognitive impairment with machine learning analysis of MRI and CSF biomarkers

**DOI:** 10.1186/1745-6215-12-S1-A18

**Published:** 2011-12-13

**Authors:** Javier Escudero, John P Zajicek, Emmanuel Ifeachor

**Affiliations:** 1 Signal Processing and Multimedia Communications Research Group, School of Computing and Mathematics, Plymouth University, Plymouth, PL4 8AA, UK; 2 Clinical Neurology Research Group, Peninsula College of Medicine and Dentistry, Plymouth University, Derriford, Plymouth, PL6 8BX, UK

## Objectives

There is a need for techniques to conduct Clinical Trials (CTs) in Alzheimer’s Disease (AD) and Mild Cognitive Impairment (MCI) more efficiently to reduce their duration and cost. However, large variability in the rating scales increases the number of subjects required to obtain significant results in the CTs [1,2]. Alternatively, Magnetic Resonance Imaging (MRI) and Cerebrospinal Fluid (CSF) are promising AD biomarkers but none is optimal for all disease stages [1,2]. Additionally, Machine Learning can detect biomarker patterns to characterize AD and MCI. In this study, we assessed the usefulness of Machine Learning to select subjects with the clearest signs of the disease for inclusion in more efficient CTs [3,4].

## Methods

We tested three Machine Learning classifiers: Logistic Regression (LR), Support Vector Machine (SVM) and Radial Basis Function (RBF) [4]. These techniques were trained to recognise disease patterns in 91 AD, 178 MCI and 106 cognitive normal (CN) subjects from ADNI [1] for whom baseline age, MRI hippocampal volume, MRI entorhinal cortical thickness, CSF Aβ_42_ and CSF phospho-Tau_181p_ levels were measured. From the classifiers, we obtained a likelihood value that each subject was AD or MCI and not CN. Then, the patients with higher likelihood (i.e., clearer signs) of the disease were first selected for inclusion in hypothetical CTs. This approach was evaluated in the terms of reduction in the number of patients needed in the CTs to detect a 25% reduction in the hippocampal volume after one year (80% power, two-sided test, *p*-value=0.05) [3,4].

## Results

Without the selection of subjects based on the classifiers, the hypothetical CTs required 109 patients for AD and 183 subjects for MCI per group (treatment vs. placebo). In contrast, the sample sizes decreased considerably when the classifiers based on the biomarkers were used to select one third of the subjects with the highest likelihood (clearest signs) of the disease, as shown in Table 1. All these group sizes were at least eight times smaller than those estimated when ADAS-cog, instead of the hippocampus, was the outcome measure in the CT (Table 2).

**Table 1:** Minimum number of subjects required per arm for a hypothetical CT with the hippocampal volume as outcome measure in AD and MCI. Two cases are considered: when all subjects are included in the CT, and when only the 33% of the subjects with the clearest signs of AD are selected for the CT.

Condition	Subset	LR	SVM	RBF
AD	All subjects	109	109	109
	33% of subjects with the clearest signs of AD	48	93	30

MCI	All subjects	183	183	183
	33% of subjects with the clearest signs of AD	95	139	104

**Table 2:** Minimum number of subjects required per arm for a hypothetical CT with the ADAS-cog as outcome measure in AD and MCI. Two cases are considered: when all subjects are included in the CT, and when only the 33% of the subjects with the clearest signs of AD are selected for the CT.

Condition	Selection	LR	SVM	RBF
AD	All subjects	1330	1330	1330
	33% of subjects with the clearest signs of AD	1675	1605	259

MCI	All subjects	8878	8878	8878
	33% of subjects with the clearest signs of AD	3098	1148	2119

## Conclusion

The results highlighted the potential of CSF and MRI biomarkers and Machine Learning classifiers (particularly LR and RBF) as objective tools to select subjects for more efficient CTs in AD and MCI. However, further analyses are needed to corroborate these results and extend this approach to other biomarkers and classifiers.
